# Surgical Principles in the Management of Lung Neuroendocrine Tumors: Open Questions and Controversial Technical Issues

**DOI:** 10.1007/s11864-022-01026-3

**Published:** 2022-10-21

**Authors:** Debora Brascia, Giuseppe Marulli

**Affiliations:** Thoracic Surgery Unit, Department of Organ Transplantation and Emergency, University Hospital of Bari, P.zza Giulio Cesare, 11 70124 Bari, Italy

**Keywords:** Neuroendocrine tumors, SCLC, Carcinoids, Surgery, Multidisciplinary treatment

## Abstract

Primary neuroendocrine tumors (NETs) of the lung represent a heterogeneous group of malignancies arising from the endocrine cells, involving different entities, from well differentiated to highly undifferentiated neoplasms. Because of the predominance of poorly differentiated tumors, advanced disease is observed at diagnosis in more than one third of patients making chemo- or chemoradiotherapy the only possible treatment. Complete surgical resection, as defined as anatomical resection plus systematic lymphadenectomy, becomes a reliable curative option only for that little percentage of patients presenting with stage I (N0) high-grade NETs. On the other hand, complete surgical resection is considered the mainstay treatment for localized low- and intermediate-grade NETs. Therefore, in the era of the mini-invasive surgery, their indolent behavior has suggested that parenchyma-sparing resections could be as adequate as the anatomical ones in terms of oncological outcomes, leading to discuss about the correct extent of resection and about the role of lymphadenectomy when dealing with highly differentiated NETs.

## Introduction

Primary carcinoid tumors of the lung are relatively uncommon, accounting for 1−2% of all lung cancers [1] and for 20–25% of all carcinoid tumors [2]. An increase in their prevalence over the last years (+6% per year) has been observed, probably due to improved awareness of the physicians to this topic and to the improvement of the immunochemical and histological identification [3]. The 2021 WHO classification categorizes NETs as a group including low- and intermediate-grade typical carcinoid (TC) and atypical carcinoid (AC), respectively, and the neuroendocrine carcinomas (NECs), including small-cell lung cancer (SCLC) and large-cell neuroendocrine carcinoma (LCNEC) [4].

Surgical resection is the mainstay treatment for localized typical carcinoids and atypical carcinoids; given their indolent behavior and their long doubling time, in the last years, their surgical management has been widely debated, questioning (1) the adequacy of sublobar resections compared to the traditional lobectomy for selected early stage cases; (2) whether lymph node involvement can be acknowledged as an independent prognostic factor; and (3) whether a radical mediastinal lymphadenectomy should always be recommended.

NECs have high mitotic rates and necrosis, thus being aggressive and starting to disseminate early in the course of the disease, with 5-year survival rates of 15–57% [5, 6]. High-grade NETs represent most neuroendocrine lung tumors, with LCNEC and SCLC accounting for 2–3% and 15–20% of all lung cancers, respectively, and are characterized by similar behavior, clinical conditions, and prognosis. In this setting, primary surgical resection is a feasible option only in the early stages, which means 10–20% of the patients with high-grade NETs; the lack of randomized controlled trials on the role of surgery still makes this approach underused and underestimated.

This review of the literature aims to analyze the role of surgery in the context of all NETs of the lungs and to summarize the most recent evidence about their correct surgical management.

## Typical and atypical carcinoids: to each patient his own resection

Among well-differentiated NETs, TCs is the most common with a TCs/ACs incidence ratio of 10:1 [7]. Histologically, TCs are characterized by less than two mitotic figures per 2 mm^2^ viable tumor and no evidence of necrosis. Moreover, they are associated with lower risk of lymph node (LN) metastases and increased survival compared with atypical carcinoids [8•]. Surgical resection is the mainstay treatment for localized low- and intermediate-grade pulmonary carcinoids, assuring a 5-year survival rate of about 90% for TC and of 70% for AC [1, 2, 9–14]. Whether the extent of surgery could influence survival is still under debate. Atypical carcinoids, because of their more aggressive nature, are treated along with the principles of NSCLC surgery: anatomic lung resection privileging lobectomy and systematic lymph-nodes dissection should be the treatment of choice in patients fit in terms of cardio-respiratory reserve. The question about the extent of surgical resection remains open for TCs [15, 16]. Due to their indolent behavior and endobronchial growth, parenchyma-sparing resections have often been advocated for TCs. The extensive review on lung carcinoid tumors by Detterbeck et al. [1] recommends assessing the optimal management for patients based on two main criteria: tumor location (peripheral or central) and N stage.

### Peripherally located TCs

**Fig. 1:**
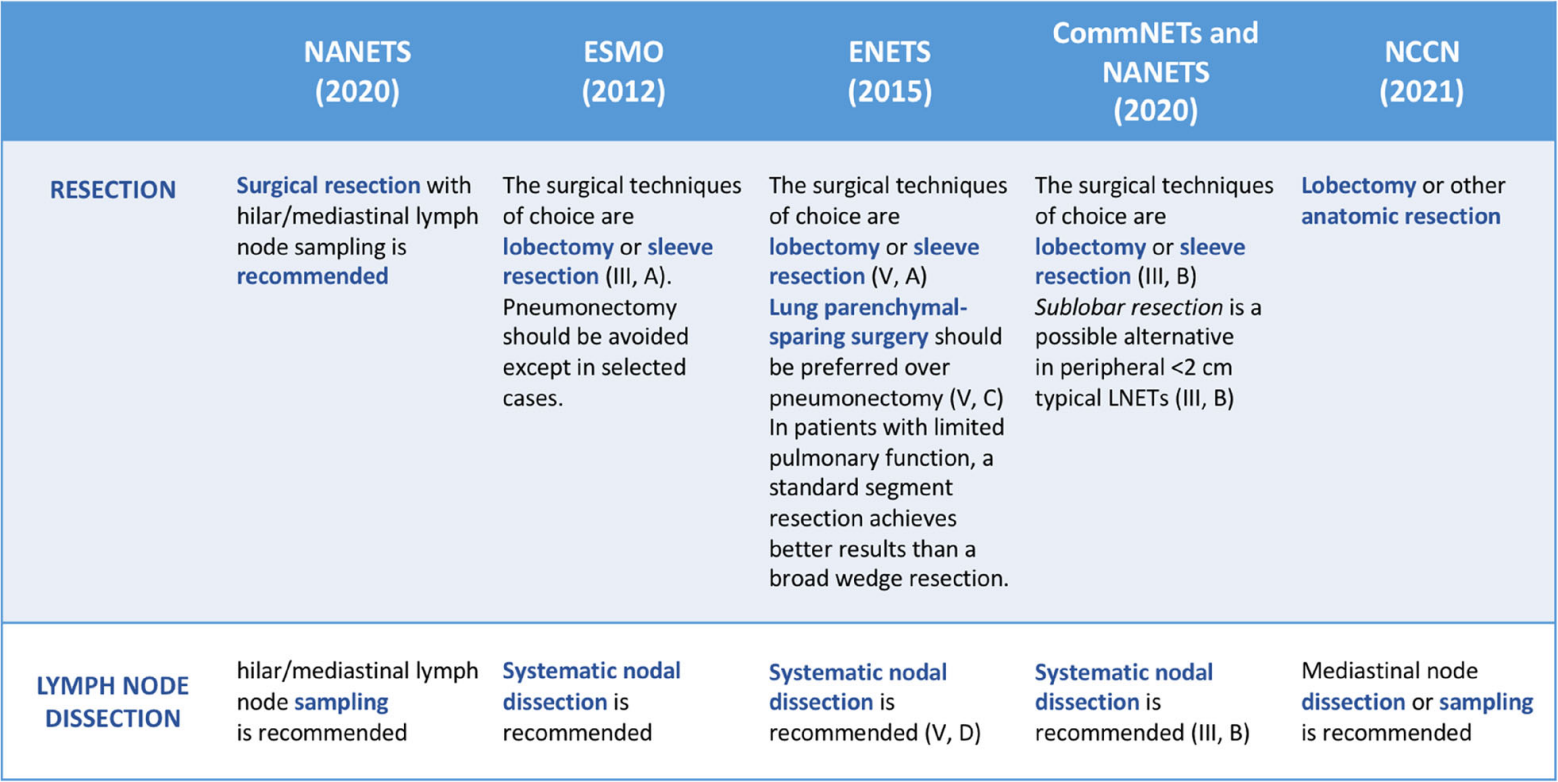
Summary of the latest guidelines on typical bronchopulmonary carcinoid management.

One of the greatest issues when discussing about the proper extent of resection for peripheral stage I TCs is that the available data are mostly derived from small, single-institution, retrospective studies; in fact, considering the relative rarity of these tumors, prospective studies are lacking and, by now, no randomized controlled trial exists targeting this topic. Figure 1 summarizes the guidelines available on this subject [2, 12, 17••, 18••, 19••].

All of them advocate for a radical anatomic resection (R0) to be obtained through lobectomy. In the last years, the Commonwealth Neuroendocrine Tumour Research Collaboration (CommNETs) and the North American Neuroendocrine Tumor Society (NANETS) by updating the 2015 European Neuroendocrine Tumor Society Expert Consensus Guidelines (ENETS) suggested that the sublobar resection may be an acceptable option in patients with peripheral typical carcinoids less than 2 cm [9]. Sublobar resections include both wedge resections and segmentectomies, without having randomized trials to favor a specific approach. Most studies, in fact, include not only both typical and atypical histologic subtypes, but also all clinical and pathologic stages as well as the entire variety of operations from wedge resection to pneumonectomy. This makes it difficult to compare the oncological outcomes of TCs only by the extent of resection. In Table 1, we summarized the latest published studies addressing the question of appropriateness of sublobar resection for resectable TCs. All studies involving both ACs and TCs were excluded. Brown et al. [8•] compared the outcomes of sublobar resection, including wedge resection and segmentectomy, with those of lobectomy for patients with clinical stage T1aN0M0 TCs.

**Table 1:** Studies reporting the outcomes by extent of resection of TCs over the last 10 years. Difference of values in **bold** is statistically significant

Author	Year	Country	Type of study	No.	Type of surgery (%)					OS per type of surgery				Sublobar vs Lobar (*p*-value)
					NoS	W	S	L	SL	NoS	W	S	L	
Brown *et al*.[8•]	2017	USA	R	1495	-	35.8		64.2	-	-	87.0	88.0		0.3
Furqan *et al.*[21]	2018	USA	R	1289	18.8	31.5		49.7	-	56.5	84.2	94.1	87.9	0.209
Filosso *et al.*[22]	2019	ITA	R	876	-	13.9	8.6	70.3	7.2	-	82	94	96	**<0.001 (W vs L)** **<0.001 (W vs S)**
Yan *et al.*[24]	2019	China	R	1887	12.4	24.6	6.0	57.0	-	73	90	94	94	0.256 (W vs L) 0.399 (W vs S)
Cattoni *et al.*[25]	2019	ITA	R	177	-	30.5	11.3	58.2	-	-	92.9		95.1	0.26
Bachman *et al.*[26•]	2021	USA	R	821	-	82.5	17.5	-	-	-	83.8	84.9	-	0.613

*R* retrospective, *NoS* no surgery, *W* wedge resection, *S* segmentectomy, *L* lobectomy, *SL* sleeve lobectomy, *OS* overall survival

In the Surveillance Epidemiology End Results (SEER) database, they found no differences in 5-year overall survival (OS) rate for lobectomy (88%) and sublobar resections (87%). Interestingly, Brown et al. draw their attention on the influence the extent of the resection plays on lymph node dissection. Sublobar resection, in fact, prevents an extensive dissection of N1 nodes, boosting the risk of missing N1 nodes between the site of wedge resection and the dissected N2 nodes. In their study, in fact, they found a higher LN upstaging in patients who had undergone lobectomy (5.2%) versus those who had undergone sublobar resections (0.7%). For this reason, anatomical resections are still preferred by some surgeons, given the propensity of TC tumors to metastasize to LNs in approximately 15% of the cases [16] with rates of 10% positivity in N2 lymph nodes verified after systematic node dissection [20]. Furqan et al. [21] also used data from the SEER database to investigate the optimal surgical management of TCs proving surgery (but not the extent of surgery) to be associated with better survival. In this study, like the previous one by Brown et al., the major limitation was that, when comparing survival rates, the authors grouped wedge and segmental resections together. Theoretically, from an oncological point of view, being segmentectomy an anatomical resection it could be considered an adequate treatment for primary lung cancers, whereas wedge resection is not. Filosso et al. [22] have overcome this limit, directly comparing outcomes of patients treated through wedge resections vs segmentectomies vs lobectomies, proving a substantial difference in terms of 5-year OS, favoring the latter two types of resections (82% vs 96% vs 94%; *p* < 0.001) and suggesting that segmentectomy should be preferred over wedge resection for a more oncological adequacy. The one by Filosso is the only study which reliably proved the superiority of anatomical resections (lobectomy or segmentectomy) over wedges for patients with stage I typical carcinoids. However, some authors have attributed those results to the higher comorbidities and older age of the wedge resection group vs the other two groups [23]. Yan et al. [24] found out that TC patients at the localized stage would benefit equally from lobectomy or sublobectomy; moreover, wedge resection proved to be equal to segmental resection in terms of survival and the choice between the two procedures seemed not to influence the prognosis of patients. Cattoni et al. [25] investigated recurrence and overall survival of patients undergoing sublobar resection vs lobectomy for clinical T1-3 N0 M0 peripheral TC of the lung. The propensity scores matched analysis showed no difference in recurrence-free survival (92.4% vs 100%; *p*=0.57) and overall survival (92.9% vs 95.1%; *p*=0.26) between patients undergoing sublobar resection and patients undergoing lobectomy. Bachman et al. [26•] directly compared wedge resections and segmentectomies for stage I typical bronchopulmonary carcinoids, proving no significant differences in 3-year and 5-year OS, 90-day mortality, length of hospitalization, and positive margin on both unadjusted and propensity-matched analysis between the two operative approaches.

Recently, the European Society of Thoracic Surgery (ESTS) Neuroendocrine Tumor Working Group (NET-WG) proposed a prognostic model to predict OS of patients undergoing surgical resection for TC [9]. They identified increased age, male gender, the presence of previous malignancies, peripheral tumors, TNM stage, and ECOG performance status as the major factors associated with mortality. Surgical approach and extent of surgery proved not to be an independent predictor of OS for patients with stage I TCs.

Previous results, although sparse and heterogeneous, prove that sublobar resection may be a reasonable alternative for resectable peripheral stage I TCs, suggesting, however, that the patient selection may influence and, consequently, should guide, the extent of parenchymal resection. In fact, older patients with a prior history of malignancy and reduced performance status with a small peripheral lesion may be best suited to undergo sublobar resection as it provides a balance with other risks. Considering the potential of sublobar resection to prevent an extensive dissection of N1 nodes, boosting the risk of missing N1 nodes between the site of wedge resection and the dissected N2 nodes, for all other patients, anatomic resections should be preferred also for stage I TCs.

### Centrally located TCs

Approximately two thirds of all carcinoid tumors arise in the major airways, and almost 20% of them presents as purely intraluminal endobronchial lesion without involvement of the lung parenchyma [1]. This peculiarity makes these tumors amenable to bronchoplastic resection, which can achieve the preservation of the lung, especially in case of poor lung function, without compromising the oncological result of the surgery, although requiring considerable technical expertise.

In this setting, endoscopy plays a major role not only for diagnosis which becomes feasible in case of endoluminal neoplasms, but also in the correct preoperative planning and in determining the feasibility of a bronchoplastic procedure [27]. Some experiences have been described in the past about the endoscopic management of these tumors with curative intent, achieved by using different endoscopic techniques such as Nd-YAG laser, diathermy, and cryosurgery [28–32]. After the first exciting and promising results, those experiences have been almost completely abandoned [33], because of the high risk of local recurrence (up to one third) after endoscopic resection, due to the tendency of TCs to spread extraluminally (iceberg phenomenon) [34]. These procedures should only be considered in selected patients with completely endoluminal growth and very small base of implant or, alternatively, for those unfit to undergo surgical resection or in a palliative setting for tumors located in the central tracheobronchial tree.

Given the indolent behavior of TCs, and their long average doubling time (close to 7 years) [35], bronchoplastic resections with/without parenchymal resection could offer a definitive solution for purely endobronchial carcinoids, even accepting minimal resection-free margins to achieve the complete control of the disease. Being these procedures technically demanding, to be performed only in high selected patients and Centers, up to date, only a few surgical series and anecdotical case reports have been published [36–42]. Raz et al. [43] performed an analysis using data from the SEER database, comparing parenchyma-sparing bronchial sleeve resections with lobar resections (including lobectomies, sleeve lobectomies, bilobectomies and pneumonectomies). They did not find any difference between the two approaches for TCs in terms of OS and recurrence, while a significant difference in OS was evident when surgical group was compared with the non-surgical one, confirming the impact of surgery on survival.

Nowak et al. [39] analyzed their results on 13 patients who underwent a parenchyma-sparing bronchial sleeve resection, with systematic nodal dissection for endobronchial carcinoid tumor without extension beyond the bronchial wall, proving that although challenging, this procedure can be carried out with very low morbidity and mortality. They observed no regional recurrence, except in a patient who presented a metachronous tumourlet in the contralateral airway after 5 years, indicating the importance of long-term follow-up for carcinoids. Rizzardi et al. [34] reported on a series of 70 patients who underwent either sleeve resections or bronchoplastic procedures for centrally located TCs, all achieving good oncological results, in the absence of local recurrence even in cases with reduced margins of healthy tissue and in two cases with microscopic infiltration of the bronchial section. Dell’Amore et al. [44] published a recent series of 98 patients treated with total-lung-sparing-tracheo-bronchoplasty for low-grade tracheo-bronchial tumors (including adenoid cystic carcinoma, inflammatory myofibroblastic tumor, typical and atypical carcinoids). Although being this procedure technically demanding, it had assured an overall 5-year survival of 97% without significant distinctions between the histology or the type of surgery performed; particularly, disease recurrence had never occurred in patients with carcinoid tumors, even in case of N-positive disease or R1-bronchial resection margins.

Bronchoplastic and parenchyma-saving procedures are safe techniques for highly selected patients with typical endobronchial carcinoids to be performed in experienced Centers; they guarantee both good oncological and functional results even with minimal resection-free margins; this evidence is provided by few retrospective surgical series and still requires further studies.

### Role of lymphadenectomy in carcinoid tumors

Due to their indolent behavior, in the past, lymphadenectomy for TC and AC has been rarely performed and the prognostic role of lymph node (LN) involvement has been addressed only by few studies. Moreover, all articles before 2000 are inaccurate since they include carcinoids according to the old classifications, causing an overlapping of the different forms of these neoplasms [1].

Nodal disease incidence in published studies ranges from 10 to 17% in TCs, almost always affecting N1 nodes, and from 30 to 64% for AC patients [10, 11]. The prognostic impact of lymph node disease has been well documented for patients with ACs [45], along with its higher incidence, and for this reason radical lymphadenectomy has been highly suggested for patients with ACs [46]. Chen et al. [46••], in fact, in their recent study focusing electively on 1033 ACs patients from the Surveillance, Epidemiology, and End Results (SEER) database showed that older age, no regional lymph nodes examination, wedge resection, pneumonectomy, lymph node metastases, and distant metastases were independent prognostic factors of worse survival, confirming the important role of lymph-node dissection (LND) in these neoplasms. A different approach has been advocated for patients with TCs, for whom the low incidence of nodal metastases and the lack of scientific evidence supporting the clinical advantage of performing a complete lymphadenectomy have long justified the avoidance of LND or the preference for just a LN sampling [11, 48]. In fact, in published series, up to one fourth of patients with small TCs had no surgical lymph node assessment performed [8, 49] while, on the other hand, some studies report a nearly 10% N2 positivity in cases of resected TCs in which a systematic node dissection had been performed [20]. Cardillo et al. [11] in a study published in 2004 reported on a series of 163 patients surgically treated for TCs and ACs with lung resection plus radical mediastinal lymphadenectomy. They found a significant difference in LN metastasis incidence (N1 and N2 metastases rates were 11.6% and 0% in TCs vs 42.8% and 21.4% in ACs) and in the overall 5-year survival between TCs and ACs (98.6% vs 70.1%) proving that this difference in survival was more related to the N status than to the histologic subtype. In fact, in N0 patients, the 5-year survival was 100% either in TC or AC; in N1 patients, the 5-year survival between TC and AC showed a non-significant difference (90.0% versus 78.8%; *p* = 0.4) while nine patients with N2 status (all of them belonging to the AC subtype) had a 22.2% 5-year survival explaining the difference between typical and atypical bronchial carcinoids. This evidence, although scarce, suggests that lymph node dissection (LND) should be regularly done when dealing with lung NETs and current guidelines (Table 1) recommend at least either lymph node sampling or dissection during the resection.

The question of whether routine sampling of N2 LNs is acceptable for early-stage typical carcinoid tumors remains open and implies further investigations. Brown et al. [8•] found that whether LN assessment was performed at the time of surgery, it did not independently predict overall survival. Conversely, LN upstaging was an independent predictor of overall survival, suggesting that sampling LNs should always be performed, as LN upstaging is associated with decreased survival. Kneuertz et al. [50] performed a study on 3335 patients with both typical and atypical carcinoids collected from the national cancer database (NCDB) and they found out that LN metastases were associated with worse survival in larger TC tumors of size greater than 2 cm but had no prognostic significance in patients with smaller TCs who underwent an adequate LN dissection. Recently, Thakur et al. [51], published their result on a series of 241 patients affected by both TCs and ACs proving that lymph node involvement is a factor of high prognostic value for disease recurrence (*p*=0.022, HR: 3.18), along with sublobar resection (wedge or segmentectomy; *p*<0.001, HR: 6.89) and atypical histology (*p*<0.001, HR: 9.89). Considering the recent evidence of the positive lymph node ratio (PLNR: defined as the ratio of positive to examined lymph nodes) to be a prognostic determinant in gastric, small intestinal and pancreatic NETs [52–55], Chen et al. [56] performed an analysis on 1662 patients from the SEER database to further evaluate the potential prognostic value of PLNR and determine the optimal LND extent. They found a PLNR ≥ 13% to be significantly associated with worse OS (*p*<0.001) and worse cancer-specific survival (*p*<0.001) than a PLNR < 13%, suggesting the PLNR ≥13% as a cut-off value to predict prognosis.

The evidence of nodal involvement when dealing with bronchial carcinoids carries significant implications. Firstly, lymphadenopathy on pre-operative imaging should be an alarm bell for the clinician, suggesting the atypical nature of the tumor, thus being more aggressive in its oncological behavior and requiring a more aggressive surgical approach. Secondly, almost all the studies, despite being heterogeneous in the patient selection, retrospective and observational in their nature and small in their sample sizes, suggest that LN involvement may impact survival both in TC and AC patients and LND (or at least a sampling for TCs) should always be performed in these cases. Further randomized controlled studies should be performed to confirm the sparse evidence and to unquestionably address this issue.

## High-grade NETS: is there a role for surgery?

High-grade NETs represent most neuroendocrine lung tumors and, due to their similar clinical behavior, most studies and large case series are focused either on LCNEC or SCLC. They are characterized by early and fast diffusion, often presenting as central and bulky lesions, vena cava compression or by early dissemination to regional lymph nodes and distant sites [57, 58].

This behavior explains the scarce prognosis of these tumors, being the worst among all lung cancers with two-year survival rates of about 5% and median survival ranging between 15 and 20 months [59]. Their nature also justifies the necessity of a systemic therapeutic approach with chemotherapy and chemoradiation, which, after the initial response to treatments, are affected by significant recurrence rates, up to 30–50% [60]. Only 10–20% of the patients with high grade NETs initially present with early-stage disease and can be treated with curative intent.

### Surgical approach for SCLC

The role of surgery in the multidisciplinary treatment of limited-stage SCLC remains unclear due to controversial literature results and the absence of recent randomized clinical trials. Historically, three randomized controlled trials [61–63] addressing the impact of surgery on survival in SCLC (performed more than 30 years ago) have led to firm ideas against surgery among clinicians up to now, and their results have also been collected in a recent meta-analysis performed in 2018 [64•]. This review concluded that there was no role for surgical resection in the management of limited-stage small-cell lung cancer, though few limitations have to be acknowledged: a) the use of older and less effective radiotherapy and chemotherapy-regimens before the advent of concurrent chemoradiation, b) the inclusion of only central tumors, c) the use of old staging modalities and, d) the enrollment of patients with either N1 and N2 disease [65].

Current guidelines recommend surgery only in those patients in stage I, who have a solitary nodule, no hilar or mediastinal involvement based on adequate mediastinal staging, no distant metastases, and no contraindications to surgery [66, 67••]. NCCN guidelines [68], in fact, recommend adjuvant chemotherapy after lobectomy and lymph node dissection even in the case of N0 disease and sequential or concurrent chemo and radiotherapy in N+ disease. These guidelines have, however, been based on limited data and, to date, there is no recent prospective study having been published evaluating surgery versus concurrent chemoradiation for early-stage nodes-negative SCLC, yet. Most of our knowledge about prognosis and survival of patients with resected SCLC comes from large retrospective population databases [69–73] including patients with various stages of disease, which have suggested an improved outcome with surgery, reopening the surgical path for patients with SCLC.

The International Association for the Study of Lung Cancer (IASLC) Lung Cancer Staging Project [69] in 2009 analyzed data on 349 cases of resected SCLC reporting 5-year survival rates as high as 48, 39, and 15% for operated patients in stage I, II and III, respectively. Similar results were those reported by analysis of the Surveillance, Epidemiology and End Results (SEER) database [70] in 2010, reporting 5-year survival rates of 50.3% for patients with stage I SCLC who underwent lobectomy without radiotherapy (RT) vs 57.1% for those who received adjuvant RT. Yang et al. [73] reported the outcome on a series of 1574 stage I SCLC patients whose data were collected in the National Cancer Database, receiving radical resection. Five-year survival rates were 47.4% for the entire cohort, ranging from 52.7 to 40.4% based on the delivery of adjuvant chemoradiation treatments.

Although these studies accounted large sample sizes, they were extremely heterogeneous, limited by the lack of chemotherapy data available, using old systemic protocols and mostly including patients who had had a diagnosis of SCLC only at postoperative examination and for this reason not being useful to give conclusively answers on the real impact of surgery on survival.

In recent years, in the light of these wide-range studies, a renewed interest in the surgical treatment of patients with stage I SCLC (T1-2 N0) has raised, and a number of studies, although retrospective, have been published precisely addressing the impact of surgery on survival, in comparison to non-surgical approaches. Table 2 summarizes main results from these studies. As evident, in the group receiving surgery, 5-year OS rates ranged from 28.0 to 62.3% vs 16.7% to 40.1% in patients treated with chemoradiation; while median OS ranged from 35 to 61.7 months for patients who underwent surgery vs 19.0 to 31.2 months for those who did not. In all cases, differences between the two groups were statistically significant, proving there was a general agreement across studies that surgery resulted in a significant OS benefit compared with nonsurgical approaches.

**Table 2:** Most recent studies reporting the survival rates of surgical vs non-surgical patients with stage I (N0) high grade lung NETs. Difference of values in **bold** is statistically significant

Author	Year	Histology	Type of study	No.	Treatment	No.	Stage I (n)	5yOS (%)	Median OS (mo)	Median OS (*p*-value)
Yang *et al*.[73]	2017	SCLC	R	2301	S + ADJ CRT	681 1620	2301	**48.1** **28.3**		
Wakeam *et al.*[75]	2017	SCLC	R	4178	S + ADJ CRT	2089 2089	1310		**38.6** **22.9**	
Xu *et al.*[96]	2019	SCLC	R	1524	S + ADJ CRT	337 1187	812		**46.0** **19.5**	
Du *et al.*[97]	2019	SCLC	R	1707	S + ADJ CRT	294 294	302			**<0.01**
Uprety *et al.*[76]	2019	SCLC	R	3879	S + ADJ CRT	749 3130	3879		**61.7** **31.2**	
Chen M *et al.*[98]	2019	SCLC	R	138	S + ADJ CRT	69 69	N/A	**62.3** **40.1**		
Zhong *et al.*[99]	2020	SCLC	R	152	S + ADJ/NEO CRT	50 102	152	**28.0** **16.7**		
Wang *et al.*[100]	2020	SCLC	R	2246	S + ADJ CRT	618 1628	457		**35.0** **19.0**	
Chen X *et al.*[77•]	2022	SCLC	R	710	S + ADJ CRT	355 355	668	**55.0** **23.0**		
Gu *et al.*[80•]	2018	LCNEC	R	704	S NS	597 107	569			**<0.001**
Raman *et al.*[85]	2019	LCNEC	R	6092	S SBRT	3222 149	3371	**50.0** **27.0**	**60.0** **35.0**	
Deng *et al*.[101]	2019	LCNEC	R	2097	S NS	392 50	442			**<0.001**
Lo *et al*.[89]	2020	LCNEC	R	39036	S SBRT	2971 238	6661	**48.0** **25.0**	**57.2** **34.6**	
May *et al*.[90]	2021	LCNEC	R	1523	S SBRT	666 82	748			**<0.001**
Sun *et al*.[102]	2021	LCNEC	R	5068	S NS	394 60	454			**<0.001**

*R* retrospective, *S* surgery, *ADJ* adjuvant chemo/chemoradiotherapy, *CRT* definitive chemoradiotherapy, *5YOS* five years overall survival, *OS* overall survival, *mo* months, *NS* non-surgery, *SBRT* stereotactic body radiation therapy

A recent meta-analysis by Stokes et al. [74] was published with the aim of assessing the outcomes of treatment strategies for limited- and extensive-stage SCLC by analyzing studies published between 2015 and 2020. In the group of limited-stage disease (including patients with stage I to III), the proportion undergoing surgery was 4.2 to 10.8%; nearly all studies demonstrated significant survival benefits favoring treatment approaches including surgery; however, no significant improvement in OS following surgery was seen in patients with stage II–III SCLC, confirming the leading role of surgical therapy only in patients with early-stage tumors.

Similar results were those described by Wakeam et al. [75] who matched 2,089 patients enrolled in the National Cancer Database; they proved surgery to be associated with longer survival for Stage I, while differences were attenuated for Stage II and IIIA.

Yang et al. [73] in 2017 published the first study evaluating the outcome of surgery with adjuvant chemotherapy versus concurrent chemoradiation for clinical T1-2N0M0 SCLC, proving improved survival outcomes for node-negative SCLC submitted to surgery, thus corroborating the current guidelines. Moreover, they also highlighted a significant underuse of surgery among patients with early-stage SCLC, likely related to old beliefs and heritages, thus supporting a leading role of surgery in multimodality therapy for cT1-2N0M0 SCLC.

Uprety et al. [76] in their retrospective study analyzed the median OS of patients who underwent surgery (70.6 months) over definitive concurrent chemo-radiation (31.2 months) in SCLC, proving a significant survival benefit in the first group. Moreover, they also evaluated the impact of prophylactic cranial irradiation (PCI) in addition to surgical approach, proving that surgery followed by chemotherapy with PCI was associated with better median OS than without PCI (93.0 months *vs* 61.7 months).

Chen et al. [77•] paired 355 early-stage SCLC patients recorded in the SEER database to analyze the role of surgery and cancer-specific survival; interestingly, they compared the survival advantage by different surgical approaches, proving lobectomy to be superior to sublobar resections and pneumonectomies, thus being considered a protective factor and an indicator of better survival in limited SCLC.

The aggressive nature of NECs of the lungs imposes an aggressive therapeutic approach in which surgery, to be accomplished through lobectomy, could have a leading role at least in the early N0 stages. In this setting, a renewed importance of early screening in SCLC should be considered since patients with stage T1-2N0M0 should undergo surgery as soon as possible to improve the prognosis. Evidence supporting these statements still need the confirmation from randomized studies.

### Surgical approach for LCNEC

Histological differentiation between LCNEC and SCLC is challenging on small biopsies and intraoperative frozen sections because of their common histological features, including the neuroendocrine morphology, large zones of necrosis and high mitotic rate [78]. For this reason, in most cases, LCNECs are diagnosed postoperatively by surgical specimens. These difficulties in the diagnosis of LCNEC, along with its rarity, have discouraged the performance of large prospective randomized trials, and, up to date, our knowledge on their best treatment strategy still relies on small sized retrospective studies. Current guidelines suggest radical surgery as the recommended treatment for patients with early-stage, resectable pulmonary LCNEC [79••] achieving 5-year survival rates ranging from 27 to 67% [80•, 81–84], although being affected by a high incidence of recurrence after radical surgery especially within the first 2 years of follow-up [78, 82]. In fact, adjuvant chemotherapy has been proved to give benefits on survival and recurrence across all stages [85], improving surgical survival outcomes even in the early stages, advocating for a multimodal approach of these aggressive neoplasms [65, 83, 84, 86–88].

Recent studies have proved surgery to add a survival benefit compared to non-surgical approaches not only in stage I LCNECs (Table 2), similarly to SCLC, but also in stage II/III LCNECs.

Gu et al. [80] in their large recent retrospective study on a total of 2594 LCNEC cases extracted from the SEER database found that surgery, when feasible, significantly improved OS compared to non-surgical treatment either when analyzing stages I–II (*p* < 0.0001), stage IIIA (*p* = 0.001), and even stage IIIB (*p* = 0.017). Similarly, Raman et al. [85] collected data on a total of 6092 LCNEC patients identified using the National Cancer Database (NCDB). Of them, 3371 patients were diagnosed as stage I and were treated with surgery (96%) or with stereotactic body radiation therapy (SBRT) (4%), proving surgery to favor OS (5-y OS 50% vs 27%, HR 0.7). Also in this study, they conducted a subgroup analysis on 736 patients with cT1–3N2M0 LCNEC, of whom 278 (38%) underwent chemoradiation and 458 (62%) surgery with perioperative chemotherapy. Both the unadjusted and the multivariable analysis proved surgery to be associated with improved survival compared to chemoradiation (HR 0.68; 95%CI 0.56–0.82; *p*<0.001). Similar results are those found by Lo et al. [89] and May et al. [90] who both compared the outcomes of surgery vs SBRT in stage I patients with LCNEC, confirming surgery as the mainstay of therapy in early-stages large cells neoplasms. Different studies have also addressed the question about the proper extent of resection, by comparing lobar vs sublobar resections in stage I LCNEC. Results from dated studies, like that by Veronesi et al. [59] who compared 15 sublobar resections with 95 lobectomies and that by Filosso et al. [91] who compared 24 sublobar resections with 81 lobectomies not reporting any difference in survival, had suggested the extent of surgical resection to be not a determinant prognostic factor. In the last years, this opinion has been criticized and reviewed since the above-mentioned results come from small single-institution sample size studies, both including patients who were stage I through III. More recent studies, in fact, agree on the fact that lobar resection is associated with improved survival compared to wedge resections and segmentectomies for stage I LCNEC [92–94, 95•], and for this reason must always be preferred.

In conclusion, surgery represents the mainstay treatment for resectable LCNEC, including stage I and stages II–III as part of a multimodal treatment plan; lobar resections should always be preferred over the sublobar ones and, in the light of the aggressive behavior of these tumors and of the high incidence of recurrence after radical surgery even in early stages, a multidisciplinary approach including a perioperative chemo-radiotherapy should always be planned.
